# Conceivability and possibility: some dilemmas for Humeans

**DOI:** 10.1007/s11229-017-1346-7

**Published:** 2017-02-24

**Authors:** Francesco Berto, Tom Schoonen

**Affiliations:** Department of Philosophy, Institute for Logic, Language and Computation (ILLC), University of Amsterdam, Oude Turfmarkt 141-147, 1012 GC Amsterdam, The Netherlands

**Keywords:** Conceivability and possibility, Imagination, Modal epistemology, Mental representation, Mental imagery

## Abstract

The Humean view that conceivability entails possibility can be criticized via input from cognitive psychology. A mainstream view here has it that there are two candidate codings for mental representations (one of them being, according to some, reducible to the other): the linguistic and the pictorial, the difference between the two consisting in the degree of arbitrariness of the representation relation. If the conceivability of *P* at issue for Humeans involves the having of a linguistic mental representation, then it is easy to show that we can conceive the impossible, for impossibilities can be represented by meaningful bits of language. If the conceivability of *P* amounts to the pictorial imaginability of a situation verifying *P*, then the question is whether the imagination at issue works purely qualitatively, that is, only by phenomenological resemblance with the imagined scenario. If so, the range of situations imaginable in this way is too limited to have a significant role in modal epistemology. If not, imagination will involve some arbitrary labeling component, which turns out to be sufficient for imagining the impossible. And if the relevant imagination is neither linguistic nor pictorial, Humeans will appear to resort to some representational magic, until they come up with a theory of a ‘third code’ for mental representations.

## Hume’s other principle

A venerable philosophical tradition makes of conceivability a main device of modal epistemology. Hume is perhaps the most quoted authority:

Tis an establish’d maxim in metaphysics, that whatever the mind clearly conceives includes the idea of possible existence, or in other words, that nothing we imagine is absolutely impossible. (*Treatise*, I, ii, 2)

As remarked by Yablo (1993), p. 4, in spite of that ‘in other words’ it is doubtful that Hume is giving the same maxim twice. It is one thing to say that, when we (clearly) conceive something, what is conceived comes by default with the embedded idea that it could exist. It is another thing to say that we can only imagine the possible. The latter claim will be our target. We call it ‘Hume’s Other Principle’ (HOP) throughout (‘Hume’s Principle’ was already taken to label a different one), and we call Humeans the supporters of HOP.

The purpose of this paper is to argue that HOP is false. This has already been done several times (e.g., Byrne 2007; Fiocco 2007; Jago 2014; Kung 2014; Priest 2016). What is, hopefully, new, is the idea that cognitive psychology can help to fine-tune the notions of conceivability and imagination, which are relevant for the assessment of HOP.

The other notion involved in HOP, absolute possibility, is nowadays reasonably under control, after the Twentieth Century development of the techniques of modal logic and possible worlds semantics. We just take the totality of possible worlds, across which all and only the genuine possibilities are represented: to be absolutely impossible is to hold at none of them.^1^

Conceivability and imagination, instead, are in a messy state. These are highly ambiguous notions, and we think that part of the debate on HOP is marred by equivocation.^2^ We also think that the large amount of empirical work carried out by cognitive psychologists on the subjects of mental representation and imagery may help to advance the discussion. But in spite of the growing interaction between philosophy of mind and cognitive science, the philosophers debating on HOP seem to us to often pay little attention to the contribution of psychology. (There are notable exceptions: see e.g. the contributions in the recent volume King and Kung (2016), in particular such papers as Langland-Hassan (2016) and van Leeuwen (2016).) We are aware that one should make cautious use of such work: it may not be clear what those empirical results tell us, given that, as we are about to see, cognitive psychologists themselves disagree on what to make of them. Still, we believe that one can extract from carefully discussed research in the area some help with our issue.

We will deal with what happens in the mind of a subject who conceives or imagines that *P*: that John kisses Mary, that Obama is blond-haired, that the Nazis have the A-bomb in 1944. We will understand these as situations in which the subject has a mental representation, whose content is *that* *P*. (It is usually said that the contents of such mental states are propositions; but we will see that the term ‘proposition’ is often used differently in cognitive psychology.) We will, thus, assume that there are such things as mental representations, which have intentionality: they are about configurations of objects and properties, situations, or circumstances, which make for their contents. To conceive or imagine that Obama is blonde-haired involves the having of a mental representation, whose content—that which the representation represents—is *that Obama is blonde-haired*. Mental representations, then, have semantic features. They can be evaluated for truth and falsity.^3^

*How* do mental representations represent whatever it is that they represent? Let us look at answers provided by cognitive psychologists.

## Mental representation: linguistic versus pictorial

At the beginning of the Chapter on the concept of representation of his seminal book, *Mental Representations*, Allan Paivio claims:

[H]istorically, mental representations have been interpreted by analogy with physical representations [...]. The most obvious distinction is that some physical representations are *picture-like* and others are *language-like*. Picture-like representations include photographs, drawings, maps, and diagrams. Language-like representations include natural human languages as well as such formal systems as mathematics, symbolic logic, and computer languages. (Paivio 1986, p. 17)

Thus many cognitive psychologists, continues Paivio, have piggy-backed on this fundamental distinction. They have understood mental representations as being of two kinds, the linguistic and the pictorial. Some have disagreed: they have claimed that mental representations represent in just one of these two ways (we will come to this at the end of this Section). Still, there is broad consensus that these are the two plausible candidates for being ways in which mental representations represent.

How do they differ? Well, how do the corresponding physical representations differ?

A consensus seems to be developing around the idea that the fundamental distinguishing dimension is the degree of arbitrariness of the mapping relation between the form of the representation and the form of the represented world. Thus, the terms picture-like, analogue, iconic, and isomorphic all imply that such representations map onto represented objects in a nonarbitrary way. In the case of language-like representations, on the other hand, the relation is completely arbitrary. (Paivio 1986, Ibid)

Philosophers will remember Peirce’s distinction between icons and symbols: icons represent via physical similarity with the relevant objects; pictures are paradigmatic icons. Symbols represent by arbitrary convention, or law; words are paradigmatic symbols. Paivio also has it that arbitrariness comes in degrees. This will be important for the discussion of HOP to come; so we inspect the point a bit further.

Take a drawing on a sheet of paper which works as a little map, representing that a river flows east to west and there’s a tree with a round-shaped crown of leaves north of the river, by having a blue line running from the left to the right of the sheet, and above it a shorter brown line oriented bottom–up, with a green circle on top of it. There is *some* arbitrariness in how the map represents. Understood conventions dictate that top, bottom, right and left stand for north, south, east and west. But there is also non-arbitrary representation secured by (relevant) similarity: that the trunk of the tree is brown and the leaves are green is represented by similarly colored parts of the sheet; that the leaves of the tree form a more or less spherical shape on top of its trunk is represented by the round shape of the green patch. Such spatial and chromatic similarity is lacking in linguistic representation. One can represent the situation in English via the sentence: ‘There’s a tree with a round-shaped crown of leaves to the north of a river which flows from east to west’. It is conventional that ‘brown’ stands for the color brown: we could have labeled that color, ‘yellow’.

Pictorial mental representations are gathered under the rubric of ‘mental imagery’, and characterized by reference to perception. Scholars speak of ‘quasi-perceptual experience’ for them (Thomas 2014, Introduction), for they resemble perceptual representation but can occur in the absence of the actual stimuli. Studies on neuroimaging such as Ganis et al. (2004) seem to show that visual mental imagery activates about 90% of the same cerebral areas activated by visual perception.^4^

Pictorial (visual) mental representations are often claimed to have, in a sense, spatial features: when we entertain imagery of this kind, we represent objects and situations typically in three-dimensional egocentric space and, as Paivio (1986), p. 198 notes, such representations are available for ‘parallel processing’ because they have some kind of mereological structure. One can, for instance, pictorially represent to oneself the arrangement of one’s own living room and describe its contents from different viewpoints, mentally scanning the objects included there from top to bottom, or from left to right, mentally zooming into a corner, etc.

Of course, psychologists who take pictorial mental representations seriously do not claim that such mental images represent by being really pictures. Hence the frequent use of the ‘quasi-’ prefix again. A claim to the effect that parts of a pictorial mental representation correspond to parts of the represented scenario, and so do relative distances, often comes with the proviso that ‘part’ and ‘distance’ should be understood functionally rather than strictly spatially (see e.g. Kosslyn and Pomerantz 1977).

Some evidence for such quasi-spatial features of mental imagery was gathered since the 1970s, thanks to widely discussed experimental work by Shepard, Kosslyn, and others on the mental rotation and scanning of mental images (Shephard and Metzler 1971; Kosslyn 1980; Pinker 1980). Such work showed, for instance, that the time taken to scan between two points of a mental image is often proportional to their subjective distance in the pictorial representation; that larger objects ‘fill’ the space sooner than smaller ones; that the level of detail of the depicted situations decreases at the periphery of the image, similarly to what happens with our visual field.

Linguistic mental representation, instead, is arbitrary in the way the connection between bits of language and what they mean is, in general, arbitrary. Psychologists often talk of *propositions* in this context.^5^ ‘Propositions are like natural-language statements that correspond semantically to external objects and events’, and are ‘completely amodal abstract conceptual structures that represent information’ (Paivio 1986, p. 31). Linguistic mental representations are called ‘amodal’ to stress that they are disconnected from sensory modalities in a way pictorial representations are not. Also, according to Paivio (1986), p. 198, they are not processed in parallel, but rather serially, the way we allegedly process the meanings of sentences through those of their subsentential components. This is taken as evidence that linguistic representations lack the mereological and quasi-spatial features of pictorial ones.

Paivio’s own *dual coding* theory, described as ‘one of the most influential theories of cognition this century’ (Marks 1996, p. 433), has it that there are precisely two codes for mental representations: the linguistic and the pictorial. Cognition works with two functionally independent (though interacting) systems handling representations of the two kinds. The usefulness of having two systems, according to some, lies in the different contents the two are apt to represent: pictorial imagery is more suitable for concrete situations which are proximal in space and time, whereas linguistic representation works better for abstract scenarios involving non-perceptual features (Amit et al. 2009).

While many cognitive psychologists understand mental representations as being of such two kinds, some deny that the kinds are really two: they variously reduce one, the pictorial, to the other, the linguistic. This involves the so-called ‘imagery debate’, or ‘analog/propositional debate’. It is, possibly, the most classical and intractable controversy on the nature of mental imagery, so something needs to be said about this.

Foes of mental images usually do not deny their existence, for mental imagery is a familiar phenomenological experience for most people in all cultures (see e.g. Marks 1999). However, they aim to show that such imagery can be variously reduced to, or that it is just an epiphenomenon of, non-pictorial, linguistic representation, lacking autonomous cognitive and functional significance. Here is one classic statement:

The *propositional representation* position on the nature of imagery has been proposed by J. Anderson and Bower (1973, p. 451 ff.), Pylyshyn (1973), and Simon (1972), and is related to proposals made by Schank (1972), Palmer (1975), and others. This position is based on the argument that all knowledge, regardless of its source modality, can be expressed in a single uniform, abstract, type of representation, the *proposition*. Unlike the dual-code position, there is no fundamental difference in how perceptually based and verbally based information is represented in memory. (Kieras 1978, pp. 533–534)

The main critic is probably Zenon Pylyshyn. His objections range from accusing the pictorial view of mental imagery of committing the homunculus fallacy by hypostasizing the metaphor of the ‘mind’s eye’, to stressing the difference between the cognitive impenetrability of early perceptual processing and the manipulability of mental representations (Pylyshyn 1973, 2002), to claiming that the supposed quasi-spatial properties of some mental imagery can always be explained away as (propositionally) encoded as information in tacit knowledge (Pylyshyn 1981). That mental representations must be essentially linguistic has also been pushed in the famous argument by Fodor and Pylyshyn (1988) against connectionism:^6^ thought must be systematic, that is, it must have a recursive structure; so mental representations must be systematic; but because connectionist models lack structured internal representations, they are bad models of thought.

We will come back to connectionism, and how it can deal with recursive representations, in a footnote below. In *this* paper, we grant to pictorialists that there is such a thing as an irreducibly pictorial way of mentally representing: the pictorial nature of mental imagery is not just an epiphenomenon, functionally reducible to the linguistic.^7^ The reason is not that we favor Paivio or Kosslyn’s views on the topic over those of Pylyshyn et al. Rather, we take such stance for the sake of the argument in our discussion of HOP. As we are about to see, if mental representations only represent linguistically, HOP cannot even get off the ground. If, on the other hand, there are such things as irreducibly pictorial mental representations, things get more complicated.

## Conceiving as having a linguistic representation

Suppose conceiving that *P*, of the kind that entails the absolute possibility of *P* according to Humeans, is to be understood as having a linguistic mental representation whose content is *that* *P*. Suppose, too, that linguistic mental representations have at least the same representational power as the expressions of natural languages like English. Call this the Parity Assumption: whatever content is representable by a natural language sentence, is also representable by some linguistic mental representation.

If linguistic mental representations are understood just as natural language sentences tokened in the head, the Parity Assumption is obvious. But even if one claims that the relevant representations are more deeply encoded, say, in a (by hypothesis, unconscious) Fodorian language of thought (Fodor 1975), one should grant that whatever content can be represented in natural language can also be represented in mentalese, given that the latter is (again, by hypothesis) supposed to ground the learnability and mastering of the former.

If the Parity Assumption is right, then the claim that we can conceive the impossible under this reading of ‘conceive’ is very plausible. To deny it, one would seem to be forced to make one of two moves: (1) claim that sentences of ordinary languages like English, describing alleged absolute impossibilities, actually are meaningless strings. Or, (2) claim that although those sentences are meaningful, and so by the Parity Assumption we can have corresponding, meaningful, linguistic mental representations, we cannot understand these even if they are tokened in our own heads.

But claim (2) is incredible in the face of the compositionality of learnable languages. Let *P* be any simple, intelligible sentence of English, such as ‘This table is round’. Surely *P* cannot become unintelligible because we stick a negation in front of it. So $\lnot P$ must be intelligible, too. And surely two such sentences cannot deliver an unintelligibility once we conjoin them, $P \wedge \lnot P$. So the latter must be intelligible, too, and by the Parity Assumption we can have a corresponding linguistic mental representation which will be intelligible in its turn, and whose content is *that* $P \wedge \lnot P$. But (unless one is a dialetheist: see Priest 1998; Berto and Priest 2013), contradictions are true in no possible world.

So we are left with claim (1). Someone who came *close* to making it is Wittgenstein (1922). We say ‘came close’, because for Wittgenstein’s *Tractatus* tautologies, logical truths, and their negations, logical falsities, are notoriously *sinnlos* (4.461). They ‘say nothing’ (Ibid.). Even for Wittgenstein they ‘are, however, not senseless [*unsinnig*]’ but ‘part of the symbolism in the same way that “0” is part of the symbolism of arithmetic’ (4.462). There is a debate among wittensteinians, on what the difference between *sinnlos* and *unsinnig* amounts to, but we don’t need to get into this. One straightforward interpretation of the Wittgensteinian view in the contemporary terminology of possible worlds, is that the informative job of a sentence is to split into two the totality of worlds: those in which the sentence is true and those in which it is false. The former group gives the proposition expressed by the sentence in standard possible worlds semantics. But then tautologies and their negations, being true everywhere and nowhere in the modal space respectively, don’t split, and turn out to be uninformative: ‘I know, e.g., nothing about the weather, when I know that it rains or it does not rain’ (4.461).

Even if one buys the view that logical truths and falsities are uninformative,^8^ though, that does not make them contentless. Even if the distinction between saying and showing at the core of the *Tractatus* is right (and some, including perhaps the later Wittgenstein, may doubt it), that, given a meaningful *P*, $P \vee \lnot P$ and $P \wedge \lnot P$ show something about the logical form of reality without informing us of what obtains in it, does not make them meaningless strings devoid of any content. Quine makes the point of the meaningfulness of contradictions in *On What There Is*, as a response to fictional philosopher Wyman, sometimes taken as representing Meinong’s view that some things do not exist (see Berto 2012). Wyman believes that things like Pegasus ought to be admitted in our ontological catalogue, as *possibilia*, for otherwise it would make no sense to even say that Pegasus is not. By parity of reasoning, objects Quine, we ought to admit the round square cupola on Berkeley College; otherwise, it would make no sense to even say that *it* is not. But accepting this brings inconsistency. Wyman reacts by declaring that inconsistent conditions are meaningless. We find Quine’s reply spotless:

Certainly the doctrine [of the meaninglessness of contradictions] has no intrinsic appeal; and it has led its devotees to such quixotic extremes as that of challenging the method of proof by *reductio ad absurdum* – a challenge in which I sense a *reductio ad absurdum* of the doctrine itself.

Moreover, the doctrine of meaninglessness of contradictions has the severe methodological drawback that it makes it impossible, in principle, ever to devise an effective test of what is meaningful and what is not. It would be forever impossible for us to devise systematic ways of deciding whether a string of signs made sense – even to us individually, let alone other people – or not. For it follows from a discovery in mathematical logic, due to Church [1936], that there can be no generally applicable test of contradictoriness. (Quine 1948, pp. 34–35)^9^

One may still object as follows.^10^ In the view under attack, conceiving *P* is bearing a certain relation, call it *C*, to a linguistic mental representation *S*, which means *that P*. In a familiar metaphor, it is to have a representation *S* which means *that P* in one’s ‘conceiving box’. Granted, there are impossible linguistic representations (by the Parity Assumption). But can we bear *C* to them? Surely a corresponding ‘belief box’ model should not be committed to the view that a cognitive agent, *x*, can believe (have a ‘*B*-relation’ to) an impossibility, just as it shouldn’t be committed, say, to the believability by *x* that *x* itself does not exist. Even when ‘I do not exist’ is (suppose) a meaningful mentalese sentence, that doesn’t mean it can be in *x*’s belief box when ‘I’ picks out *x*. As an analogy, take a bulletin board on which announcements can be pinned. It may be a well-enforced rule that no political flyers can be attached to the board even though the content of the flyers is perfectly meaningful and intelligible. Something could prevent ‘I do not exist’ from being in one’s belief box; and similarly for the conceiving box.

However, this analogy between a conceiving and a belief box should be resisted. It is generally agreed by scholars who work on propositional attitudes that conceiving (and, imagining) work(s) very differently from believing in relation to what can get into the respective boxes. In particular, as argued by Wansing (2015) and Langland-Hassan (2016), conceiving, just like *supposing*, is subject to voluntary control in ways believing is not. Conscious acts of conceiving often have an *arbitrary* starting point. This may be made up by the agent (‘Now let’s imagine what would happen if...’), or it may be given as an external instruction (think of going through a novel, taking the sentences you read as your explicit input as you revise your imagined scenario). If conceiving only involves the having of a linguistic mental representation, we can generally put in our ‘conceiving box’ what we can put in our ‘box of suppositions’, even if we just can’t put it in our belief box. For instance: one can conceive (or, imagine) that London has been displaced to France but, having overwhelming evidence of the contrary, one cannot make oneself believe it by pure will. Similarly, one can conceive, or imagine, not to exist (*x* imagines a world without *x* in it) although one cannot believe it.

A plausible explanation for why this is so is that conceiving—again, understood as the having of a linguistic mental representation—just like supposing, is neutral in ways believing is not: believing requires commitment, which is absent when one just supposes (see Balcerak Jackson 2016 on this point). Similarly, it would be pragmatically inconsistent to assert ‘I do not exist’, but it is not pragmatically inconsistent to advance the claim as a supposition (‘Imagine my parents had never met, so I was never born; then you would probably have ended up marrying John...’). The attitude is one of allowing a certain content to show up for consideration, not taking a stance on its being realized.

In their influential book on mental simulation, Nichols and Stich (2003) make the point explicit in terms of mental boxes. For Nichols and Stich, mental simulation works via what they call a ‘possible worlds box’, where we voluntarily put ‘an initial premiss or set of premisses, which are the basic assumptions about what is to be pretended’ (p. 24). This box, for Nichols and Stich, is connected to our ‘belief box’ because we integrate the explicit pretense’s content with a selection of our beliefs. However, they make clear that the two do not coincide and ought not to be confused, for we can bear the *C*-relation to lots of things we cannot bear the *B*-relation to. And in spite of their speaking of ‘possible worlds’, the explicit premise that makes for the starting point of our acts of mental simulation can well be impossible:

We are using the term ‘possible world’ more broadly than it is often used in philosophy [...], because we want to be able to include descriptions of worlds that many would consider impossible. For instance, we want to allow that the Possible World Box can contain a representation with the content *There is a greatest prime number*. (Nichols and Stich 2003, p. 28)

## Conceiving as imagining

If conceiving is understood merely as the having of a linguistic mental representation, we can conceive the impossible; for we can conceive contradictions, thus, HOP is false. In fact, as Christopher Hill has remarked, in this sense—which he dubs ‘simple, undisciplined conceiving’, ‘virtually anything is conceivable’, and ‘conceivability is therefore incapable of providing a reliable test for possibility’ (Hill 2016, p. 326).

However, philosophical discussions around HOP, e.g., in such works as those of Hill (1997) himself, but also Stoljar (2007) and Kung (2010), and various essays in the collection Gendler and Hawthorne (2002), seem to take into account a sense of ‘conceiving’, which *may* involve something else than having a linguistic mental representation. Chalmers speaks of ‘positive conceivability’:

Positive notions of conceivability require that one can form some sort of positive conception of a situation in which *P* is the case. One can place the varieties of positive conceivability under the broad rubric of *imagination*: to positively conceive of a situation is to imagine (in some sense) a specific configuration of objects and properties. [...] Overall, we can say that *P* is positively conceivable when one can imagine that *P*: that is, when one can imagine a situation that verifies *P* (Chalmers 2002, p. 150, notation modified.)

Similarly, Yablo (1993) has it that the notion of conceivability which is relevant for Humeans amounts to the imaginability of a world verifying *P* (Yablo grants that we do not imagine the relevant world in all detail). However, philosophers have often characterized such notion only by pointing at some features making it differ from other intentional states (e.g., that imagining, unlike believing, is subject to voluntary control in certain ways; that it does not involve commitment to the actuality of the envisaged scenario, etc.), doubting that one can do much better in this regard.^11^

These characterizations do not deliver a clear answer to the key question: *What kind* of mental representation is involved in such exercises of imagination? Authors sympathetic to HOP, like Chalmers and Yablo, have little to say on this. We heard Chalmers speaking of positive conceivability as ‘imagining (in some sense)’ a specific configuration of objects and properties. After singling out the relevant kind of imagination, Yablo claims, in a footnote:

Some philosophers use ‘imagine’ so that imagining a thing is *imaging* it, that is, conjuring up an appropriate sensory presentation. I do not require a sensory-like image for imagining, and certainly not a distinct such image for distinct imaginings. (Yablo 1993, p. 27)

Now if we take seriously the framework we have derived from our survey in Sect. 2, our question can be phrased as: Is the mental representation involved in the kind of imagination which, according to Humeans, entails possibility, pictorial mental representation, or not?

Supposing it is not, as Yablo’s answer seems to suggest, the next question is: Is it linguistic, or is it some other kind of mental representation which is neither linguistic nor pictorial? If the former, we are back to the case of Sect. 3, and there is little hope for HOP.

However, this may not be the preferred answer. Various philosophers stress that imagining that *P* should be understood as a mental operation different from merely supposing or assuming that *P*, as when we make an assumption in a mathematical proof, in spite of the fact that both, unlike believing that *P*, do not involve commitment to the actuality of the imagined content. Tamar Gendler claims that imagination ‘is also sometimes distinguished from mental states such as conceiving and supposing, on the grounds that imagining S requires some sort of quasi-sensory or positive representation of S, whereas the contrasting states do not’ (Gendler 2011, Introduction; see also Gendler 2000; Kind 2001; Balcerak Jackson 2016). If ‘merely supposing or assuming’ is taken as the tokening of a linguistic mental representation, this would indicate that the relevant imagining is not to be understood as involving only linguistic mental representations.

But if the involved representations are taken as being neither linguistic nor pictorial, this leaves the Humeans with a heavy burden of proof. They seem forced to invoke a peculiar ‘third code’ of representation for the relevant imagining—one that may have no counterpart in general theories of representation, and which, above all, has no counterpart in mainstream psychological theories of mental representation. It is now up to the Humeans to provide a plausible psychological theory of the relevant imagining. It seems fair for their opponents to claim that, pending such a theory, what is being invoked is some representational magic.

Suppose, then, that the imagination relevant for HOP, possibly *pace* Yablo, *is* pictorial: it requires the having of mental imagery, pictorially representing (at some level of detail) a world making *P* true. Humeans then face another dilemma. For now the question is: Does the imagination at issue work *purely* pictorially, or not? What is meant by this question, is whether the involved mental imagery represents a world or situation where *P*, without any element of language-like, arbitrary labeling or meaning assignment, but rather, purely qualitatively: only via the phenomenological and quasi-spatial similarity of the imagery to the situation or world making *P* true.

Physical pictorial representations need not represent purely pictorially. Our drawing of the tree north of the river from Sect. 2 represents what it represents, partly by chromatic and geometric similarity between the colored areas of the sheet and the shape and color of the tree and of the river, and partly by the stipulation that *x*’s being north of *y* be represented by the representation of *x*’s being drawn above that of *y* on the sheet. Even more importantly, it represents via the stipulation that the green patch with a brown line below it represents *the tree*, and the blue line oriented from left to right on the sheet represents *the river*.

It has been argued by a number of scholars that pictorial mental representation can *never* work in cognition only by similarity, or purely pictorially: we go from Goodman (1976)’s general argument opposing the symmetry of similarity to the asymmetry of the representation relation, to Fodor’s charges of lack of compositionality and insufficient specificity for mental imagery (Fodor 1975, 1981). But assume that the criticisms don’t work; that there does exist mental imagery representing purely pictorially, with no labeling or arbitrary meaning-assignment; and that this makes for the kind of imagination involved in HOP. Then the only scenarios imaginable in this way seem to be those that involve exclusively primary and secondary perceptual qualities (colors, shapes, extension, motion) of physical objects arranged in space-time. Then we can never imagine, in the relevant sense, situations involving abstract objects, or any non-perceptual feature of concrete objects. Even if we can imagine purely pictorially that John kisses Mary, for instance (which is doubtful anyway—we will get back to this), we cannot imagine in this way that John and Mary are second cousins, for *being a second cousin of* is no perceivable feature of things.

But when philosophers discuss whether the imaginability of intrinsic universals, time travel, a supreme being having all perfections to the highest degree, entails via HOP their absolute possibility, the relevant imagination cannot be purely qualitative or pictorial, for it involves abstract objects and/or properties quite removed from sensory perception. Thus, in such debates ‘imagination’ seems to be understood more broadly than what a purely pictorial characterization of the notion can sustain. Otherwise, we would be dealing with a concept too limited to have any significant role in modal epistemology and cognition.^12^ (We don’t mean for purely pictorial imagery to have no role at all, as may be suggested by the fact that actual perception can have a role in modal epistemology: see Strohminger 2015.) As argued e.g. by Siewert (1998) and Siegel (2006), some labeling is needed whenever perceptual experience as well is to have a richer content than just primary or secondary qualities. You see a face and a nose, rather than face-like and nose-like shapes, for your experience comes labeled: the nose-like shape has to represent a *nose*, the face-like shape has to represent a *face*.^13^

Now if the kind of imagination which is relevant for HOP crucially involves labeling components, then the relevant mental representations cannot be purely pictorial mental imagery. Some elements in the imagery must work as labels for the objects and features of the represented scenarios. It will be argued throughout the rest of this paper that it is such labeling component that spells trouble for HOP, under the hypothesis—the only one remaining, if our arguing by cases has left nothing else out—that the imagination which entails possibility according to Humeans is pictorial-cum-labeling mental imagery.^14^ This will involve a detour through the ideas of a philosopher, whose work is strictly linked to the topic of HOP: Kripke. Talk of ‘purely qualitative’ representation, as opposed to ‘stipulation’, may have reminded philosophers of Kripke’s criticism of Lewisian counterpart theory and modal realism from *Naming and Necessity*. We will see that that criticism bears important connections to the issue of HOP.

## Kripkean error theory

What is Kripke’s own stance on HOP, to begin with? The post-Kripkean acceptance that, contrary to what much philosophical tradition believed, there are a posteriori necessities, is sometimes taken as hitting HOP hard. For, the argument goes, identities such as those between Hesperus and Phosphorus or between water and $\hbox {H}_{2}\hbox {O}$ are empirical discoveries. Could we then not conceive of things as being otherwise, and so conceive the impossible? It seems easily imaginable, in some sense or other, that water may have turned out to have a different chemical constitution.^15^

However, in *Naming and Necessity* Kripke proposes a different diagnosis of the phenomenon, which, according to Kung (2014), amounts to an attempt to support HOP by explaining contrary appearances away via an error theory. Kripke has little to say on the psychology of the mental representations involved in HOP. His key idea is that some imaginings are compatible with their authors’ making errors in appreciating the represented content. Specifically, such errors may involve misidentifications:

‘Heat is the motion of molecules’ will be necessary, not contingent, and one only has the illusion of contingency in the way one could have the illusion of contingency in thinking that this table might have been made of ice. We might think one could imagine it, but if we try, we can see on reflection that what we are really imagining is just there being another lectern in this very position here which was in fact made of ice. (Kripke 1980, pp. 160–161)

A posteriori necessary truths can give us an ‘illusion of contingency’: it may have turned out on empirical investigation, one thinks, that Hesperus is not Phosphorus or that water is not $\hbox {H}_{2}\hbox {O}$. Then these matters must be contingent. Kripke explains the illusion by resorting to intentional doppelgängers. We can think we are imagining a possible scenario in which water is not $\hbox {H}_{2}\hbox {O}$. What we actually imagine, though, is a situation qualitatively identical to, or indiscernible from, one we may find ourselves in, and in which we face some fluid that has the same phenomenal features of water (a colourless, odourless, tasteless liquid, etc.), without being $\hbox {H}_{2}\hbox {O}$. We can also imagine having cherished that watery stuff with the name ‘water’. But such imagining is not the representation of an impossibility, that is, of (what we actually refer to as) water not being what it necessarily has to be. The illusion comes from misjudging our own representation, misidentifying that doppelgänger of water with water.

To generalize: when we seem to imagine a situation *S* falsifying an a posteriori necessity *P*, (1) we actually imagine a qualitatively indiscernible scenario $S_{1} \ne S$, such that (2) $S_{1}$ is possible and (thus) no falsifier of *P*, and (3) we confuse $S_{1}$ with *S*.

Error theories do not have a great track record in philosophy, and we think this one is no exception. In the following Section, we will argue that the strategy does not work in all cases—as it should if, as required by HOP, we can never imagine the impossible. We will also argue that the conception of imagination underlying such error theory is wrong: it misunderstands the imagination relevant for HOP for purely pictorial mental imagery.

## The telescopic and stipulative views of imagination

According to Hill (1997), the strategy of redescribing imagined wannabe-impossibilities as imagined possibilities + misidentification ‘is fundamentally misguided’ because ‘in non-pathological circumstances introspection gives us pretty accurate access to the contents of our own states of imagination’ (p. 83); thus, it is implausible to assume, as the error theory needs to do, that we are systematically mistaken whenever we think we are imagining the impossible. But even if we were mistaken in some or even many cases, the Kripkean redescription strategy just won’t work in all cases. One example, proposed in Wright (2002), is that of first-person counterpossible imagining.

If Kripke is right, Wright claims, I am essentially a human being, and necessarily tied to my actual biological originators. But I can imagine myself as having been born from different parents. I can also imagine myself, say by putting myself at center stage in a fantasy story, as being an elf, an alien, a monkey. Can my imagining these scenarios be explained away as my imagining possible situations involving an intentional doppelgänger of mine, which I mistakenly identify with myself? It seems not, says Wright. For I do not individuate myself *qua* thinking subject by means of phenomenal, surface appearances, as I individuate water by its external appearances of colorless, tasteless liquid. When I imagine myself in a clearly possible counterfactual situation, such as my being in the Grand Canyon instead of Europe, ‘no mode of presentation of the self need feature in the exercise before it can count as presenting a scenario in which I am in the Grand Canyon’ (Wright 2002, p. 436). The same holds for my counterpossible imagining myself as a monkey: this is not easily redescribable as my imagining a doppelgänger which is a monkey, and mistakenly taking the substitute to be me. I imagine *myself* in this case as well.

Kripkean redescription also doesn’t appear to be available with mathematical conjectures and impossibilities. If we can imagine in the relevant sense mathematical conjectures whose truth value we ignore, we can, in general, equally easily imagine that they are true, and that they are false.^16^ A mathematician may imagine that Goldbach’s Conjecture fails. She may try to see what would follow from this. Suppose that the conjecture is true. If mathematical necessity is absolute, then it is absolutely impossible for some even number (larger than two) not to be the sum of two primes. Still, we cannot easily redescribe the mathematician’s imagining the relevant impossibility as the conceiving of a false doppelgänger of the content of the conjecture. What could such a doppelgänger be?

Proven conjectures, such as Fermat’s Last Theorem, make the case more vivid. Take a competent, but skeptic mathematician, who imagines she can find some mistake in Andrew Wiles’ proof, or even direct counterexamples to the theorem. The person understands the content of the theorem pretty well: it’s a simple claim on Diophantine equations. It is implausible to redescribe the situation as the mathematician’s imagining counterexamples to an intentional duplicate of the content of Fermat’s Theorem. There appears to be no content-misidentification going on here. Wright concludes from similar cases that ‘for a large class of impossibilities, there are still determinate ways things would seem if they obtained’ (Wright 2002, p. 437).

Aside from problems of generality, it seems to us that the Kripkean error theory comes, to use a label borrowed from Kung (2014), with a ‘telescopic view’ of the imagination at issue in HOP: when we imagine a scenario where *P*, we look with a metaphorical mental telescope at a situation making *P* true. What cannot happen is that such mental telescope has us look at the impossible: if the scenario *shows up*, it is *there* to be seen. What can happen is that we fail to appreciate what scenario we are looking at.

Talk of ‘telescopic view’ by Kung is meant to remind us of Kripke arguing *against* a telescopic view of our access to worlds in the aforementioned objections to Lewis. The context is the problem of transworld identification in the philosophy of possible worlds, which, as far as we know, is originally due to Kaplan (1969). This is an epistemic issue, not to be confused with the problem of transworld identity, which as far as we know is due to Chisholm (1967). The latter can itself be phrased in different ways (Divers 2002, Ch. 16, makes the relevant distinctions), but it is in any case an issue of (modal) metaphysics. The former has to do with how can we know whether we have a case of transworld identity in some sense or other.

In Kaplanian terms: which of the individuals in a possible world *w* is the ‘transworld heir’ of an individual in a different possible world (say, the actual one, @)? Given our own David Lewis at @, we are supposed to carry out some investigation among the individuals in *w*, with the aim of locating the Lewis-representative there. The problem seems intractable, insofar as *w* may include several individuals who resemble Lewis in various respects and can compete for the role. Here is one individual whose fingerprints and facial expression are indiscernible from those of our own David, but who never did philosophy and had a career as a drug dealer. Here’s another one who does not quite look like David, but who has written a book called *On the Plurality of Worlds*, where he defends the view that possible worlds are disconnected spacetimes largely filled with concrete stuff, etc.

Scholars usually consider transworld identity as a real issue (unless one is a counterpart theorist, which few want to be anyway), and transworld identification as a pseudo-problem, precisely under the influence of Kripke. This pseudo-problem comes, for Kripke, from a purely qualitative view of how worlds represent possibilities. Other worlds, says Kripke, are not something we glance at via the famous telescope. We need not represent alternative situations in purely qualitative terms: ‘generally, things aren’t “found out” about a counterfactual situation, they are stipulated.’ (Kripke 1980, p. 49), *et cetera*: the story is so well known that it hardly needs rehearsing (see also Plantinga 1974, p. 95; Chihara 1998).^17^

Now if the kind of conceivability of *P* invoked by Humeans is the imaginability of a world or situation verifying *P*, and this involves having pictorial mental representations, such representations must make for pictorial-cum-labeling imagination on pain of being of very limited use for modal epistemology. Then they work, we suggest, more like Kripkean stipulation than, as presupposed in Kripkean error theory, as a Kripkean telescope. Kung (2014) claims that its stipulative component is what gives to (what we have called) pictorial-cum-labeling imagination its power to access the impossible. In particular, the identity of the represented objects in an exercise of imagination can in general be stipulated—it does not need to be discovered.

One imagines John kissing Mary. The phenomenology of the mental imagery can be such that the represented figures are relevantly similar to John and Mary: hair color, eyes, bodies. But what makes the imagining count as a representation of a scenario in which *John* kisses *Mary* is that one takes one figure as representing *John* and the other as representing *Mary*. And just as one can imagine John kissing Mary—a possible scenario—so can one imagine John as a cleverly disguised robot: one labels the person-lookalike one is imagining, which turns out, on inspection, to be filled with circuits and transistors instead of flesh and blood, as old *John*. But the latter scenario, if Kripke is right, is metaphysically impossible.

The need for labeling of this kind can be appreciated also by considering qualitatively indiscernible imaginings which are about different scenarios. One imagines two mono-zygotic twins one knows, standing next to each other, John on the left and Paul on the right, to the level of detail in which the two are qualitatively indiscernible in the scenario. What makes the one on the left John, and the one on the right Paul, is that they are so labeled. Had the label been inverted, we would have had qualitatively identical imagery representing a different situation: one in which John is standing on the right, and Paul on the left.

Once one accepts that the identity of the objects involved in such exercises of imagination is settled arbitrarily, one easily grants the imaginability of situations where the identity of objects is other than what it actually is: one can imagine Mohammed Ali punching Cassius Clay, and Hesperus as distinct from Phosphorus.^18^

If one now retorts that it may then be in our powers to also stipulate, in imagining a world *w* that makes *P* true, that *w* be *possible*, one is then doomed to trivialize the role of conceivability-as-imagination as a tool for the discovery of possibility: for HOP can then be secured as true by definition. One is, in effect, characterizing the relevant imagination as one that entails possibility by fiat, begging the question against anyone who does not already agree with HOP to begin with.^19^

## Conclusion

To sum up: we have assessed HOP, the claim that we cannot conceive or imagine the impossible, by addressing the question of what kind of representation is involved in the relevant mental states. We have looked at theories of mental representation from mainstream cognitive psychology, and we have seen that these deliver a series of dilemmas to Humeans. If conceiving is the having of a linguistic mental representation, the Humean claim is utterly implausible. If conceiving is imagining in the sense of having of a pictorial mental representation, then either this represents purely pictorially, or it includes some labeling component. Even if purely pictorial representation is possible at all, it seems to have such a limited range of application that it is useless for modal epistemology. If, on the other hand, labeling is involved, then this makes the impossible imaginable again. And if Humean conceiving is neither pictorial nor linguistic, it is not clear what it may be.

## Footnotes

1 Philosophers debate on the nature of absolute possibility and impossibility, but it is fair to say that the three main kinds of absolute modalities are usually taken to be the logical, the mathematical, and the metaphysical. We will not get into the issue of whether one of them is reducible to another (e.g., the mathematical to the logical, as it may be for supporters of the original Hume’s Principle). We are, also, not interested in what is at times called the nomologically impossible: the impossible relative to some body of natural laws. All parties in the debate around HOP agree (Gendler and Hawthorne 2002, Introduction) that we can conceive the physically impossible, like (if Einstein was right) a body moving faster than the speed of light.

2 Stephen Yablo—to whom we owe one of the best philosophical efforts of disambiguation—claims that ‘Tigers with round-square striping are not imaginable; neither can we imagine tigers that lick all and only tigers that do not lick themselves’ (Yablo 1993, p. 30). Graham Priest replies: ‘... tigers that lick all and only those tigers that do not lick themselves. I find this no harder to imagine than a set that contains all those sets which are not members of themselves. (And I could imagine this even before I became a dialetheist.)’ (Priest 2016, p. 10). One may suspect that the two are targeting different notions.

3 Such an assumption would be challenged at the outset by intentional eliminativists like Churchland (1981), or by radical enactivists like Hutto and Myin (2013). However, it seems dialectically fair in a discussion of HOP. That there are mental representations seems to be assumed both by Humeans and by their critics. The debate on whether we can mentally represent impossible scenarios may need to be deeply recast if there are no such things as mental representations of scenarios. For a classic defense of the need for mental representations, see Clark and Toribio (1994).

4 Perception can involve different senses, thus psychologists study imagery in other sensory modalities, too: auditory, olfactory, haptic imagery. However, the visual is by far the most investigated imagery modality, and also the most relevant for our discussion of HOP. On the connections and differences between mental imagery, actual perception, and dreaming, see Ichikawa (2009).

5 And, what they mean is usually different from what most philosophers mean. They do not mean the content of sentences (as philosophers do, e.g., when they debate whether propositions are structured entities or sets of worlds), but rather the sentences themselves. Thus, to have a ‘propositional’ (in the psychologist’s sense) mental representation of a state of affairs where *P* is to have a sentence-like linguistic representation whose content is the proposition (in the philosopher’s sense) that *P*.

6 Thanks to an anonymous referee for reminding us of this.

7 There is also a recent form of converse reductionism, where essentially (multi-)modal mental imagery is taken as capable of performing most or all of the functions that traditional, computationally-minded cognitive science had ascribed to amodal, propositional representations, including reasoning and operating with abstract concepts. The work of Barsalou on so-called ‘grounded cognition’, for instance, has had a lot of influence (Barsalou 1999, 2008).

8 We do not buy this view either, because we accept a cognitive (as opposed to merely environmental) conception of information. Consider what can be learned by a rational, but finite and fallible agent—one of us. We can learn that a complex formula, whose truth value we were ignorant of until we computed its long truth table, is a tautology. For all we knew before carrying out the computation, the formula’s being false was a way things could be. In this sense, *pace* Wittgenstein (6.1251), there are surprises in logic. A book defending this view is Jago (2014).

9 A friend of true contradictions such as Graham Priest is on the same page on this: ‘If contradictions had no content, there would be nothing to disagree with when someone uttered one, which there (usually) is. Contradictions do, after all, have meaning. If they did not, we could not even understand someone who asserted a contradiction, and so evaluate what they say as false (or maybe true). We might not understand what could have brought a person to assert such a thing, but that is a different matter and the same is equally true of someone who, in broad daylight, asserts the clearly meaningful “It is night”.’ (Priest 1998, p. 417).

10 Thanks to an anonymous referee of this journal for bringing up the point. The objection is phrased as in her/his original comment.

11 ‘Almost never in philosophy are we able to analyze an intentional notion outright, in genuinely independent terms: so that a novice could learn, say, what memory and perception *were* just by consulting their analyses. About all one can normally hope for is to locate the target phenomenon relative to salient alternatives, and to find the kind of internal structure in it that would explain some of its characteristic behavior.’ (Yablo 1993, pp. 25–26)

12 One can find in Fodor (1975) the view that pictorial mental representation has a role in cognition only insofar as it is present in the form of what he calls ‘image under description’. Fodor phrases the point in terms of his underlying language of thought hypothesis, so he speaks of images endowed with mentalese labels: descriptions in the language of thought pinning down what the image is about. Tye (1991) proposes that mental imagery be taken as hybrid, mixing pictorial and linguistic ways of representing. According to Tye, mental images are ‘(labeled) interpreted symbol-filled arrays’, where the symbols represent linguistically, that is, by working as labels assigned to what they stand for; whereas the quasi-spatial arrangement of the symbols in the array pictorially represents the spatial arrangement of the objects in the imagined scenario.

13 The analogy with perception may show, as highlighted by Kung (2010), that talk of arbitrary labeling and stipulation need not mean that pictorial-cum-labeling imagination is, in general, a two-stage process: first the pure phenomenology of imagery, then the conscious, free deliberation of what the elements of the imagery are to stand for. In a sense, we cannot help but see a face and a nose when we look at a friend: it’s not that we first see those shapes and colors, and then decide that they are to mean a friendly face. What matters for our point is only that the mental imagery relevant for HOP does not represent purely by similarity, but also via the fact that elements of the imagery work as labels.

14 As one anonymous referee of this journal pointed out to us, some writers have maintained that *connectionist* representations resist classification in terms of linguistic/pictorial/mixed. This can be disputed, however. If there are representations supported by connectionist models at all, it is relatively uncontroversial that the pictorial ones qualify. As for the linguistic, the issue here is whether these models can support representations which can be combined recursively, as per the rules of a linguistic syntax. Work by Paul Smolensky on how connectionist systems can differentiate strings with the same components linearly combined in different (syntactically allowed) orders gives evidence that this is indeed the case: see Clark (2014), pp. 84–85. A similar point is made in Chalmers (1990), who exploits a technique called *recursive autoassociative memory*. If this strategy of response is right, connectionist models differ from classical architectures for cognition and AI, more with respect to how mental representations are, or can be, implemented, than with respect to how these can be classified.

15 At the time of *The Meaning of Meaning*, Putnam was resolute on this: ‘We can perfectly well imagine having experiences that would convince us (and that would make it rational to believe that) water isn’t $\hbox {H}_{2}\hbox {O}$. In that sense, it is conceivable that water isn’t $\hbox {H}_{2}\hbox {O}$. It is conceivable but it isn’t logically possible! Conceivability is no proof of logical possibility [...] Human intuition has no privileged access to metaphysical necessity.’ (Putnam 1975, p. 133).

16 One may claim that there just is no kind of mental imagery at work when we conceive such abstract scenarios as those involving only mathematical objects. Then we are back to the other horns of a dilemma in Sect. 4: this is going to be imageless, linguistic mental representation, or magical representation. Either way, the Humeans are in trouble. For an account of the role of mental imagery in mathematical reasoning, see Giardino (2010).

17 ‘There is something amiss when a claim of the type “Suppose that Socrates had never gone into philosophy...” is met with the challenge to demonstrate how you know that it is Socrates that is the object of your supposition. The same might be said of the question how you know that the subject of the proposition that Socrates is a philosopher is the same as the subject of the proposition that Socrates was married [...]. The theme that unites the last two deflationary thoughts is that one can “give” a possible world or a representational content in a non-qualitative way by relying on stipulation.’ (Divers 2002, p. 272).

18 In *Towards Non-Being*, Priest takes this stipulative feature of pictorial-cum-labeling imagination as evidence for the imaginability of impossibilities: ‘[W]hen I imagine that water is not $\hbox {H}_{2}\hbox {O}$ I am imagining something about *water*. The imagination is *de re*. In the same way, when I imagine that Sarah Palin was the US Vice President after the 2012 US election, I am imagining something about *Palin*. When I imagine that Routley found a box that was empty and not empty, it is *him* that I imagine. And when I imagine that 361 is a prime number (it isn’t) I am imagining something about that very number.’ (Priest 2005, p. 195).

19 ‘It is one question how we know which objects are the objects of our *de re* modal thought and talk, and there stipulation has a legitimate role. It is another question altogether how we know what is modally true of those objects, and there stipulation has no legitimate role to play.’ (Divers 2002, p. 273).
